# Factors Impacting Patient Satisfaction at a Single Academic Sports Medicine Institution

**DOI:** 10.7759/cureus.77732

**Published:** 2025-01-20

**Authors:** Daniel Cushman, Christian Wirawan, Nikolas Kazmers, Masaru Teramoto

**Affiliations:** 1 Physical Medicine and Rehabilitation, University of Utah, Salt Lake City, USA; 2 Orthopaedics, University of Utah, Salt Lake City, USA; 3 Physical Medicine and Rehabilitation, Schwab Rehabilitation Hospital, Chicago, USA; 4 Orthopaedics, University of Chicago, Chicago, USA

**Keywords:** patient-centered outcomes research, patient satisfaction, press ganey, quality assessment, quality assessment in healthcare, sports medicine, sports research

## Abstract

Introduction: Patient satisfaction has become an important metric in many healthcare settings, as it relates to patient outcomes and improvement in healthcare quality. The Press Ganey tool is a common assessment used to evaluate patient satisfaction. To our knowledge, there are no studies that effectively determine what influences a patient’s healthcare experience in a sports medicine setting. The purpose of this study was to determine which factors impact Press Ganey patient satisfaction at a single, academic sports medicine institution.

Methods: Press Ganey surveys with questions about the provider, appointment date, sex, age, first visits vs. subsequent visits, and in-person vs. telehealth visits were utilized and emailed to patients within one week of their visit. Questions were rated on a Likert scale from 1 (very poor) to 5 (very good). Data from 2017 to 2022 were then aggregated retrospectively and de-identified. The 2022 Area Deprivation Index (ADI; higher scores indicate higher deprivation) was also used based on the patient's zip code. Statistical analysis with the use of a multivariate linear regression model was used to identify potential variables associated with patient-reported provider scores.

Results: The study included data from 12,518 patient visits managed by eight sports medicine providers. Analysis revealed that age, appointment date (specifically the first visit), and the provider involved had statistically significant associations with Press Ganey satisfaction scores, with p-values of <0.001 for each factor. Age was positively correlated with satisfaction (coefficient = 0.160), indicating that older patients reported higher satisfaction levels compared to younger patients. Conversely, the appointment date, particularly at the initial visit, had a negative coefficient, demonstrating that patient satisfaction increased with subsequent visits. Additionally, when analyzing Press Ganey scores in relation to the ADI, it was found that patients with lower ADI scores, which indicate lower levels of social deprivation, reported higher satisfaction with their care providers.

Conclusion: Our study reveals that patient satisfaction seems to be affected by age, timing of visits, socioeconomic status, and provider at an academic sports medicine institution. While these factors may be somewhat unmodifiable, a better understanding of patient and provider characteristics can help maximize the patient's satisfaction with their healthcare.

## Introduction

Patient satisfaction has become a new metric in an attempt to improve the current healthcare system [1]. Improving a patient’s experience of care is one of the triple aims deemed important by the Institute of Healthcare Improvement [2,3]. More value is now placed on delivering healthcare that is not only clinically effective but also patient-centered [3]. Furthermore, payers, notably Medicaid/Medicare, have begun to offer value-based reimbursement that focuses on the patient’s healthcare experience [4]. Thus, providers now have increasing incentives to maximize care focused on patient satisfaction [4].

Although a provider’s intrinsic abilities and personality likely relate to a patient’s satisfaction, many extrinsic factors relate directly to how they are rated [5,6]. Age, education, sex, religion, travel distance, and wait times are just a few of the variables that have been shown to relate to patient satisfaction [7]. Knowledge of these factors could help providers identify patients who may have a lower likelihood of satisfaction. Despite the importance of patient satisfaction in healthcare improvement, a uniform approach to assessing these factors does not exist [5,6]. Patient satisfaction surveys, such as Press Ganey (PG), have become an effective solution to the gap in the literature [8]. Press Ganey satisfaction scores have been used in a number of clinical settings ranging from orthopedic clinics, emergency departments, academic centers, etc. [1,9,10]. To date, no studies have examined factors relating to patient satisfaction amongst non-operative sports medicine providers. Thus, our study will focus on factors affecting patients’ experience and satisfaction at an academic sports medicine clinic.

## Materials and methods

The Institutional Review Board approval was first obtained at the primary institution. Press Ganey scores were consecutively collected in a de-identified manner retrospectively from a single academic institution from 2017 through 2022, consisting of eight non-operative sports medicine providers. All potential patients were included if surveys were fully completed. Primary specialties of these providers included physical medicine & rehabilitation, internal medicine, emergency medicine, and family medicine, all with advanced sports medicine training. Patients had unique identifying numbers; thus, repeat visits were able to be assessed. Patients could have multiple visits with the same or different providers. Over that time period, each patient received a link by email asking them to rate their visit within a few days of visit completion. A reminder email was sent within a week if the first survey was not completed. At that point, no further survey links were sent and no responses were recorded. The Appendices list the clinical aspects that were queried. Questions were graded on a Likert scale from 1 (very poor) to 5 (very good) and converted to a 100-point scale. The total score was meant to reflect the patient’s view of their sports medicine provider and clinic.

The 2022 Area Deprivation Index (ADI) score [11] was identified for each patient based on the patient's zip code. A higher value represents more disadvantage in the area, as a percentile from 1 to 100. In addition to the ADI, potential independent variables of interest were included: provider, appointment date, first visit (as opposed to repeat visit), sex, age, and in-person (as opposed to telehealth) visits.

For the statistical analysis, means and counts were calculated from the data. The primary outcome variable was the patient-reported provider score (a higher score indicating a better rating of the provider). Univariate linear or logistic regression models were performed individually to identify potential variables relating to the primary outcome variable. Values of p ≤ 0.20 were included in the main multivariate linear regression model. Within the final multivariate regression model, a robust variance estimate was used to adjust for within-cluster correlation for individual patients [12]. Stata V17.0 (StataCorp LLC, College Station, TX) was used for analysis, with significance set at p < 0.05.

## Results

A total of 12,513 patient encounters (5,618 patients with 3,504 first-time visits) met the inclusion criteria, as seen in Table 1. Eight sports medicine physicians were included, ranging from 806 to 2,338 patient encounters (428 to 926 patients) each during the study period. Of the entire cohort, 3,365 (59.9%) were female. All subjects were composed of a mean age of 55.7 ± 16.1 years and were seen in person for 11,865 (94.8%) of the visits (the rest were virtual). They came from 442 different (five-digit) distinct zip codes and the top five nearby states (Utah, Nevada, Idaho, Montana, and Wyoming) were utilized for the ADI values.

**Table 1:** Demographic information of the patients included in the study. N = 12,513 patient encounters from 5,618 individual patients.

	Mean (SD) or n (%)
Age, years	55.7 (16.1)
Female sex	3,365 (59.9%)
Race	
White	4,917 (87.5%)
Hispanic	236 (4.2%)
Asian	124 (2.2%)
Other/unreported	339 (6.0%)

Initial univariate analyses demonstrated the following variables to potentially be related to the primary outcome variable (patient satisfaction with the provider, p ≤ 0.20): age (p < 0.001), first visit (p < 0.001), provider (p < 0.001), lower ADI (p < 0.001), and appointment date (p = 0.073). This allowed for a final regression model using those particular variables, which can be seen in Table 2. All independent variables in the model demonstrated statistical significance. Thus, older age, later appointment date, subsequent visits (as opposed to first visit), lower ADI, and provider were all related to higher patient-reported Press-Ganey scores. For example, older patients were more likely to rate their providers more favorably (0.16 (0.136, 0.185) points per year of age).

**Table 2:** Results of multivariate regression model – identifying factors associated with higher patient-reported Press Ganey scores of their provider. ADI = Area Deprivation Index; r2 = 0.0393; n = 11,209 encounters; root mean square error = 14.0.

Variable	Coefficient	Std. error	T-value	P	95% Conf.	Interval
Age	0.160	0.0126	12.71	<0.001	0.136	0.185
Appointment date	-0.00141	0.000297	-4.75	<0.001	-0.00199	-0.000823
Provider	-0.443	0.0731	-6.06	<0.001	-0.586	-0.300
First visit	-1.798	0.347	-5.18	<0.001	-2.48	-1.12
ADI	-0.0303	0.0105	-2.90	0.004	-0.0508	-0.0098
Constant	120	6.49	18.4	<0.001	107	132

**Figure 1:**
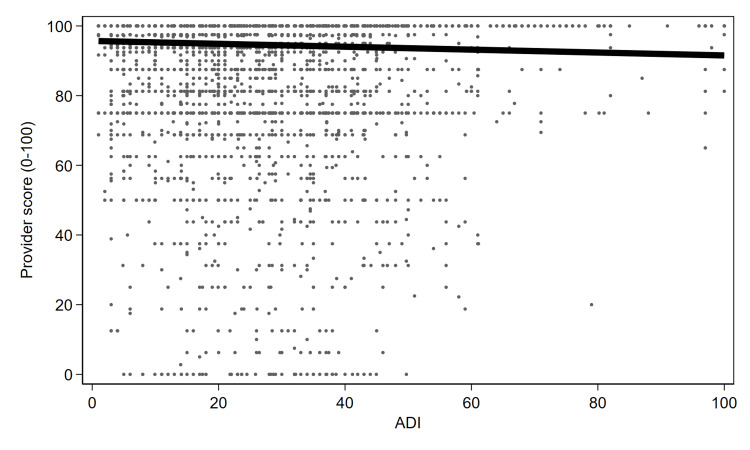
Patient-reported Press Ganey scores for the provider, as a function of Area Deprivation Index (ADI) score. X-axis = Area Deprivation Index. Y-axis = Press Ganey scores for the provider.

**Figure 2:**
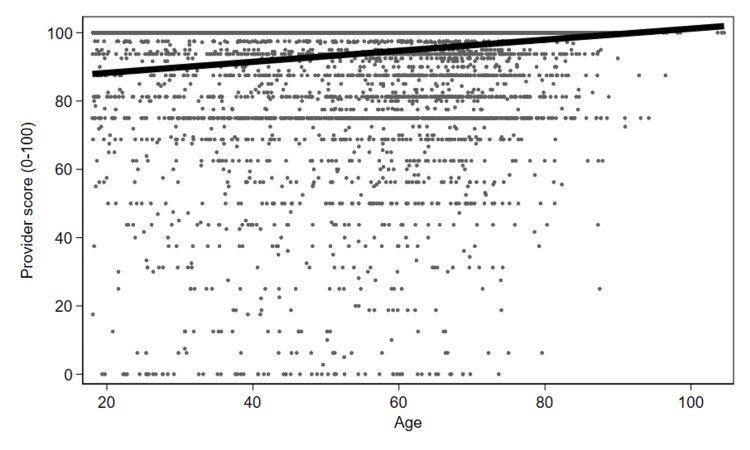
Patient-reported Press Ganey scores for the provider, as a function of patient age. X-axis = Age of the patient in years. Y-axis = Press Ganey scores for the provider.

Figure 1 displays all scores related to patient ADI scores. In conjunction with the aforementioned regression findings, this demonstrates that with increasing ADI (more deprivation), patients reported significantly lower provider satisfaction scores. Figure 2 demonstrates a similar but inverse finding for age - increasing age demonstrates significantly higher Press Ganey scores.

## Discussion

As there have been limited studies focusing on patient satisfaction within sports medicine, the purpose of this study is to identify key factors that significantly impact patient satisfaction with their provider at a single academic sports medicine clinic. The results of this study demonstrate that four factors (age, timing of visit, ADI, and the provider) significantly impact patient satisfaction for sports medicine providers at our site.

Firstly, older participants were found to have increased satisfaction compared to younger patients. Participants increasing in age were found to have statistically significantly higher Press Ganey satisfaction scores than that of their younger cohort, similar to the findings in non-sports medicine-related fields [13,14]. Although patient-related factors, such as the general perception of the physician or time availability, may relate to this difference, a paper by Peck also confirms that physicians may offer more patient-centered care to elderly patients [15], which may in turn affect patient satisfaction.

Subsequent visits also had higher patient satisfaction as compared to initial visits, as scores increased with subsequent visits. There are currently no studies comparing a patient’s first visit and their return visits within the sports medicine field. However, the results of our study are supported by similar findings within other healthcare fields, as patients at return visits were associated with higher patient satisfaction [16]. This could be related to a number of factors, as explained by a study conducted in emergency care and primary care, including but not limited to wait times, explanation of results/treatments, and help received [16-18]. The study further goes on to say that a patient’s willingness to return is largely related to their satisfaction, thus potentially explaining the higher Press Ganey scores at return visits [17]. This suggests the importance of patient satisfaction when wanting to improve continuity of care [18]. One could also hypothesize that patients’ conditions may be improving with time and treatment, and thus more likely to rate their satisfaction as higher.

Similar to a study by Stephens et al., patients with fewer disadvantages (low ADI score) had higher satisfaction when compared to more disadvantaged patients [19]. Socioeconomic status (SES) has been shown to play a role in a patient’s overall physical and mental well-being [20]. Studies have indicated that lower educational levels and average income can affect a patient’s ability to understand health concerns and ask questions [21,22]. This may suggest that patients with lower SES are less likely to participate in shared physician-patient decision-making, potentially lowering patients' satisfaction with their care [21,23]. Conversely, studies have shown that SES may be tied to a patient’s perception of the care they receive [24]. Furthermore, a systematic review by Verlinde et al. found that physicians tended to have less communication and give less explanation to lower SES patients as compared to their counterparts [21]. Thus, patients with higher social deprivation may have reason to be less satisfied with the care they receive or may represent a communication barrier.

Patient-centered care has been proven to affect patient satisfaction and our findings applied in a sports medicine setting prove no difference [25,26]; the provider that offers care significantly impacts patient satisfaction related to that provider. Both modifiable and non-modifiable physician factors affect patient perception of a visit, though these can be challenging to study. Prior studies have shown a variety of non-modifiable risk factors related to lower patient satisfaction scores, including female gender, Asian ethnicity, and being unmarried [27]. Other modifiable risk factors associated with lower patient satisfaction scores included poor communication/listening skills, poor nonverbal scores, respect for patient preferences [7], and poor technical skills [28]. This further suggests that the care a provider gives to their patients tends to have the most influence on patient satisfaction [1,29]. The results of our study identify non-modifiable risk factors (age, timing of visit, social deprivation). While the practitioner cannot change these risk factors, it may be useful to still address them. Communication at the level of the patient may be key, focusing on age, cultural values, education level, and socioeconomic background.

While prior work has focused on other settings [16-18,27], the unique setting of a sports medicine clinic may directly impact patient satisfaction. Time spent in the clinic appears to affect outcomes in this setting [29], and the relative health and lower acuity may lend toward higher outcomes compared to the emergency room setting or surgical services.

This current study is not without limitations. Since our study was conducted at a single academic institution, our results may not be generalizable to other patient populations. In addition, satisfaction surveys, such as Press Ganey, require full patient participation; survey fatigue could have impacted the quality of our results [30]. The response rate could not be calculated; thus, there is likely a response bias that was introduced. Finally, information about the patients was not readily available, such as their interests, sports, and degree of activity.

## Conclusions

In conclusion, our study is the first of its kind showcasing how patient satisfaction is impacted within the sports medicine field. As sports clinics mainly focus on patients getting back to doing activities they love, our study demonstrates that similar to other specialties, there are both modifiable and non-modifiable factors that affect patient satisfaction. Our study identified four key factors that significantly influence patient satisfaction scores: patient age, timing of the visit, social deprivation, and the physician providing care. While many elements within these factors may be difficult or impossible to modify, it is crucial for sports physicians to prioritize patient-centered care. This approach can positively shape patients’ perceptions of the care they receive, potentially improving satisfaction levels and mitigating the impact of non-modifiable risk factors. Future research should focus on modifiable risk factors that can improve patient satisfaction, in addition to how to address non-modifiable risk factors through improved communication.

## Appendices

List of clinical aspects about which patients were asked in the Press Ganey survey. The care provider score was used as the primary outcome variable for this study.

1. Overall (staff working together, likelihood to recommend practice).

2. Access (ability to get the desired appointment, ease of scheduling, registration staff courtesy, ease of getting on the phone).

3. Moving through visit (information about delays, wait time).

4. Nurse (nurse friendliness, nurse concern).

5. Personal issues (staff protecting safety, sensitivity to needs, concern for privacy, practice cleanliness).

6. Care provider (friendliness/courtesy, explanation of problem or condition, concern for questions/worries, effort to include in decisions, information about medications, follow-up care, speaking with clear language, time spent, confidence in provider, likelihood to recommend).
